# MicroRNA‐302c modulates peritoneal dialysis‐associated fibrosis by targeting connective tissue growth factor

**DOI:** 10.1111/jcmm.14029

**Published:** 2019-01-28

**Authors:** Xiejia Li, Hong Liu, Lin Sun, Xun Zhou, Xinke Yuan, Yusa Chen, Fuyou Liu, Yu Liu, Li Xiao

**Affiliations:** ^1^ Department of Nephrology The Second Xiangya Hospital Central South University Changsha China

**Keywords:** connective tissue growth factor, fibrosis, mesothelial‐mesenchymal transition, microRNA‐302c, peritoneal dialysis

## Abstract

Long‐term peritoneal dialysis (PD) can lead to the induction of mesothelial/epithelial‐mesenchymal transition (MMT/EMT) and fibrosis; these effects eventually result in ultrafiltration failure and the discontinuation of PD. MicroRNA‐302c (miR‐302c) is believed to be involved in regulating tumour cell growth and metastasis by suppressing MMT, but the effect of miR‐302c on MMT in the context of PD is unknown. MiR‐302c levels were measured in mesothelial cells isolated from the PD effluents of continuous ambulatory peritoneal dialysis patients. After miR‐302c overexpression using lentivirus, human peritoneal mesothelial cell line (HMrSV5) and PD mouse peritoneum were treated with TGF‐β1 or high glucose peritoneal dialysate respectively. MiR‐302c expression level and MMT‐related factors alteration were observed. In addition, fibrosis of PD mouse peritoneum was alleviated by miR‐302c overexpression. Furthermore, the expression of connective tissue growth factor (CTGF) was negatively related by miR‐302c, and LV‐miR‐302c reversed the up‐regulation of CTGF induced by TGF‐β1. These data suggest that there is a novel TGF‐β1/miR‐302c/CTGF pathway that plays a significant role in the process of MMT and fibrosis during PD. MiR‐302c might be a potential biomarker for peritoneal fibrosis and a novel therapeutic target for protection against peritoneal fibrosis in PD patients.

## 1 INTRODUCTION

Peritoneal dialysis (PD) is one of the most important renal replacement therapies for patients with end‐stage renal failure. However, continual exposure to non‐biocompatible PD fluid (PDF), the mechanical stress of the dwelling solution, and catheter complications (including peritonitis and haemoperitoneum) may cause acute and chronic inflammation and injury of the peritoneum. In these conditions, the peritoneum undergoes structural and functional alterations, which ultimately lead to peritoneal fibrosis and ultrafiltration failure; however, these consequences subsequently limit the long‐term clinical application of PD.^1^ Mesothelial‐to‐mesenchymal transition (MMT) is the initial and reversible process that leads to fibrosis in some organs; because MMT is a source of peritoneal fibroblasts in PD and plays a central role in the peritoneal alterations leading to fibrosis during PD, an increasing number of studies have been carried out to analyse the characteristics of MMT in PD.^2^, ^3^, ^4^, ^5^ However, the mechanism of this process is very complicated, and no effective therapy has yet been found. Therefore, it is urgent to find an effective method to investigate the mechanism and an applicable treatment for peritoneal fibrosis.

MicroRNAs are non‐coding RNAs that post‐transcriptionally regulate gene expression by inducing target mRNA degradation or inhibiting its translation. Recent studies have confirmed that miRNAs, such as those in the miRNA‐200 family, miRNA‐205, miRNA 30b, miRNA‐21‐5p and miRNA‐129‐5p, are important regulators of TGF‐β1‐induced MMT in PD.^6^, ^7^, ^8^ In our previous study, we employed a miRNA array analysis of the PMCs from the effluent of PD patients and identified aberrant miRNA levels involved in the MMT of PMCs. The results indicated that the expression of 269 miRNAs was dysregulated; of these, 124 miRNAs were up‐regulated and 145 miRNAs were down‐regulated. MiR‐302c was found to be down‐regulated nearly threefold in the PMCs from the effluents of patients undergoing PD for more than 1 year when compared with those from patients undergoing PD for less than 6 months. However, the role of miR‐302c in the MMT process of PMCs remains unknown.

MiR‐302c belongs to the miR‐302 family, which is highly conserved and plays important roles in regulating cellular proliferation, differentiation and reprogramming.^9^, ^10^ MiR‐302c was found to take part in multiple physiological and pathological processes. MiR‐302c can regulate the differentiation of human embryonic stem cells,^11^ and its effect on the formation and development of tumours has also been studied. MiR‐302c may directly target the oestrogen receptor in human breast cancer,^12^, ^13^ and it has also been reported to be dysregulated in biliary tract cancer 14 and thyroid cancer.^15^ Interestingly, it was delineated that miR‐302c was involved in idiopathic pulmonary fibrosis via regulating the transition of epithelial‐to‐mesenchymal (EMT).^16^ More recently, Kai Zhu et al^17^ revealed that miR‐302c suppressed the tumour growth of hepatocellular carcinoma through the metadherin‐mediated inhibition of EMT. These studies suggested a potential role of miR‐302c in EMT and fibrosis. Considering mesothelial cell is a kind of epithelial cell and MMT is actually a subtype of EMT, thus it aroused our interest to investigate the effect and mechanism of miR‐302c in MMT and fibrosis during PD.

Connective tissue growth factor (CTGF), a downstream mediator of transforming growth factor‐β1 (TGF‐β1), was thought to take part in TGF‐β1‐induced fibrosis and to be related to the occurrence and development of fibrosis in many organs because it is a pro‐fibrotic factor.^18^, ^19^ CTGF was also detected in the peritoneal fluid of patients undergoing PD. Some studies have revealed that peritoneal mesothelial cells can produce CTGF, which subsequently drives peritoneal fibrosis by inducing fibroblast proliferation and collagen synthesis; moreover, CTGF expressions are significantly increased in the thickened peritoneal membrane of PD patients with ultrafiltration failure (UFF). However, the exact mechanism has not been well described, especially in peritoneal fibrosis during PD. Consistent with the findings above, our previous studies have showed that the CTGF expression is up‐regulated in both PD rats and the effluent liquid from PD patients with peritoneal fibrosis; these data indicate a positive correlation between CTGF and TGF‐β1.^20^, ^21^

The goal of this study was to identify the role of miR‐302c in peritoneal fibrosis. We analysed miR‐302c expression in mesothelial cells isolated from the PD effluent from patients on PD and evaluated its relationship with markers of MMT and peritoneal fibrosis. Furthermore, we employed lentiviral miR‐302c to up‐regulate the expression of miR‐302c and then observed whether the overexpression of miR‐302c can alleviate the MMT and peritoneal fibrosis during PD and the relationship between it and CTGF. We have been suggested that that miR‐302c may inhibit TGF‐β1‐induced MMT and fibrosis by suppressing the expression of CTGF.

## 2 MATERIALS AND METHODS

### 2.1 Animal model

Male ICR mice weighing 28‐30 g were purchased from the Experimental Animal Department of Central South University and raised in the Laboratory Animal Center of second Xiangya Hospital. A mouse model of peritoneal fibrosis was induced by daily intraperitoneal injection of 1.5 mL 4.25% dextrose PD fluid (Baxter, Guangzhou, China) for 30 days. To determine the extent of MMT and duration of miR‐302c in peritoneal membrane in mouse model of PD, mice were randomly divided into normal control (D0) and PD treatment for 15 days (D15) and 30 days (D30) (n = 6 in each group). To examine the functional role of miR‐302c in PD‐induced peritoneal fibrosis, mice were randomly allocated into four groups (n = 5 in each group). Mice in group CTL received daily intraperitoneal injection of 1.5 mL 0.9% sodium chloride for 30 days; mice in group PDF received daily i.p. injection of 1.5 mL 4.25% dextrose PD fluid for 30 days; mice in groups miR‐302c and pGIPZ received daily i.p. injection of 1.5 mL 4.25% dextrose PD fluid and treatment of lentivirus containing mmu‐miR‐302c (LV‐mmu‐miR‐302c) or blank plasmid (LV‐pGIPZ) once at the day before PD. The parietal peritoneal membrane was collected for morphological examination, and visceral peritoneal membrane for protein and mRNA analysis. All animal experiments were approved by the Animal Management Committee of Central South University and performed in compliance with the university's guidelines.

### 2.2 Peritoneal equilibrium test (PET)

PET measurements were carried out as previously reported.^22^ Briefly, a 2.5% dextrose dialysate exchange was performed overnight using a PD patient's standard dwell volume. The peritoneal cavity was drained for over 20 minutes, then instilled 2.5% dextrose dialysate into the peritoneal cavity. After 4 hours, remove 10 mL effluent to determine the urea, creatinine and glucose levels. Peritoneal membrane transport function was designated as high, high average, low average or low transporter.

### 2.3 Human peritoneal mesothelial cells (HPMCs)

To determine the expression of miR‐302c in PD patients, the overnight (8 hours) peritoneal lavage of continuous ambulatory peritoneal dialysis (CAPD) patients were collected and centrifuged to isolate peritoneal mesothelial cells as previously reported.^23^ All patients involved were >18 years of age. Patients were excluded if they were >65 years old or had history of abdominal infection or malignancy. Patients with a history of kidney transplantation, haemodialysis for >3 months before PD or immunosuppressive agent use for a long time were also excluded. Ultimately, 20 PD patients were included. Newly enrolled patients receiving CAPD less than 3 months were used as controls (PD start, n = 10) and patients who were receiving CAPD for more than 1 year were used as the PD >1 year group (n = 10). MiR‐302c expression in the PMCs was detected by TaqMan probe real‐time fluorescence quantitative PCR. The study was approved by the Institutional Review Board and Ethics Committee of the second Xiangya Hospital, Central South University, China. All participants thoroughly read and then signed the informed consent form in accordance with the World Medical Association Declaration of Helsinki.

### 2.4 Lentivirus‐mediated gene transfer of miR‐302c

The lentivirus transfer vectors containing hsa‐miR‐302c (pGIPZ‐hsa‐miR‐302c) and mmu‐miR‐302c (pGIPZ‐mmu‐miR‐302c) were synthesized by Shanghai Medical and Biological Engineering Technology Company. pGIPZ as a blank plasmid contained a GFP sequence for monitoring the fluorescence expression. Lentivirus was produced by co‐transfecting human kidney 293T cells with ViraPower™ Packaging Mix plasmids (Life Technologies, Burlington, ON, Canada) and pGIPZ‐hsa‐miR‐302c, pGIPZ‐mmu‐miR‐302c or pGIPZ using Lipofectamine 2000 (Roche Biosciences, Basel, Switzerland). Cells were incubated overnight at 37°C 5%CO_2_, then the medium was replaced by 15 mL in DMEM (Gibco, Waltham, MA, USA) with 10% FBS. Cell culture supernatants were harvested 48 hours later. The supernatant was cleared by centrifugation and filtration with a 0.45 μm Millex^®^ syringe filter. Then the supernatant was pooled and layered onto 10 mL 20% sucrose solution and centrifuged at 25 000 r/min for 30 minutes. The pellets were solubilized in DMEM, the samples were fractioned and stored at −80°C until use. The lentivirus stock titrate was detected using flow cytometry to analysis the ratio of GFP positive cells. The mice received single intraperitoneal injection of 2 × 10^8^ gene transfer units (GTU) of LV‐mmu‐miR‐302c, diluted in 1.5 mL saline at the day before PD for the experiment. The HPMCs were transduced with 2.5 × 10^7^ GTU/mL LV‐hsa‐miR‐302c or LV‐pGIPZ using Polybrene.

### 2.5 Cell culture

HMrSV5 (a human peritoneal mesothelial cell line, HPMCs) was obtained from Dr Pierre Ronco (Tenon Hospital, Paris, France). Cells were grown in Dulbecco's modified Eagle's medium/F12 medium containing 10% foetal bovine serum and incubated at 37 in a humidified incubator with 5% CO_2_. In in vitro experiment, cells were incubated with 5 ng/mL TGF‐β1 (Prospecbio, Inc., East Brunswick, NJ, USA) in serum‐free medium for 0‐48 hours to investigate the effect of TGF‐β1 in the MMT change and expression of CTGF in HPMCs. In addition, with the treatment of 5 ng/mL TGF‐β1, HPMCs were transfected with LV‐hsa‐miR‐302c (LV‐pGIPZ served as a control), and with or without 10 ng/mL recombinant CTGF protein (R&D System, Minneapolis, MN, USA) to explore the precise role of miR‐302c and CTGF in TGF‐β1 induced MMT in HPMCs.

### 2.6 MiRNA microarray analysis

Total miRNA was extracted from visceral peritoneum or cells with the mirVana miRNA Isolation Kit (Ambion, Austin, TX, USA) according to the manufacturer's instructions. MiR‐302c was detected using the TaqMan^®^ MicroRNA Assay (Applied Biosystems, Foster City, CA, USA) and SYBR^®^ Premix Ex Taq™ (Perfect Real Time) (TaKaRa Bio, Inc., Beijing, China). The input was normalized by U6 snRNA. MiR‐302c and U6 primer sets were purchased from Applied Biosystems.

### 2.7 Real‐time PCR

Total RNA was extracted from visceral peritoneum or cells using Trizol Reagent (Invitrogen, Carlsbad, CA, USA) according to the manufacturer's protocol. For the detection of vimentin, Zo‐1, E‐cad, α‐smooth muscle actin (α‐SMA), col I and CTGF mRNA, total RNA was reverse transcripted to cDNA by RevertAid H Minus First Strand cDNA Synthesis Kit (Fermentas, Hanover, MD, USA) and subsequently amplified by PCR. The primer pairs used are listed in Table S1.

The real‐time PCR analyses were performed with the ABI Prism 7900 sequence detection system (Applied Biosystems). Expression was calculated using the 2^−ΔΔCt^ method and normalized to glyceraldehyde‐3‐phosphate dehydrogenase. Three independent experiments were performed, and the results were given as means ± SD.

### 2.8 Western Blot

For western blot analysis, the visceral peritoneum and cells were homogenized in RIPA buffer and then centrifugated to get proteins in the supernatant. The proteins were subjected to SDS‐PAGE gel and transferred to a nitrocellulose membrane. Protein expression was analysed by western blot analysis with primary antibody against human/mouse vimentin (Santa Cruz Biotechnology, Inc., Dallas, TX, USA), Zo‐1 (Santa Cruz Biotechnology, Inc.), α‐SMA (Santa Cruz Biotechnology, Inc.), E‐cad (Santa Cruz Biotechnology, Inc.), Col I (Cell Signaling, Inc., Danvers, MA, USA) or CTGF (Santa Cruz Biotechnology, Inc.) and then incubated with an appropriate secondary antibody. After washing, the protein was visualized and quantified by BIO‐RAD ChemiDoc™ XRS+ with Image Lab™ Software. The relative protein levels of vimentin, Zo‐1, Col I, E‐cad, α‐SMA and CTGF were normalized to β‐actin.

### 2.9 Histopathological and fluorescence examination

Paraffin sections (4 μm thick) from the anterior abdominal wall were stained with HE and Masson's trichrome for observing the morphological changes of peritoneal membrane. The thickness of the submesothelial collagenous zone was determined under microscope in coded slides using a ocular metric. The expression of green fluorescence in ICR mice peritoneum was detected under fluorescence microscope after LV‐mmu‐miR‐302c or LV‐pGIPZ transfection.

### 2.10 Statistical analysis

Data obtained from this study were expressed as means ± SD, and the statistical significance was analysed using a one‐way anova and an unpaired *t* test (GraphPad Prism, version 5.01, San Diego, CA, USA). Differences were considered statistically significant at *P* < 0.05.

## 3 RESULTS

### 3.1 MiR‐302c was down‐regulated in PMCs from the effluent of PD patients; miR‐302c expression negatively correlated with MMT and CTGF expression

**Figure 1:**
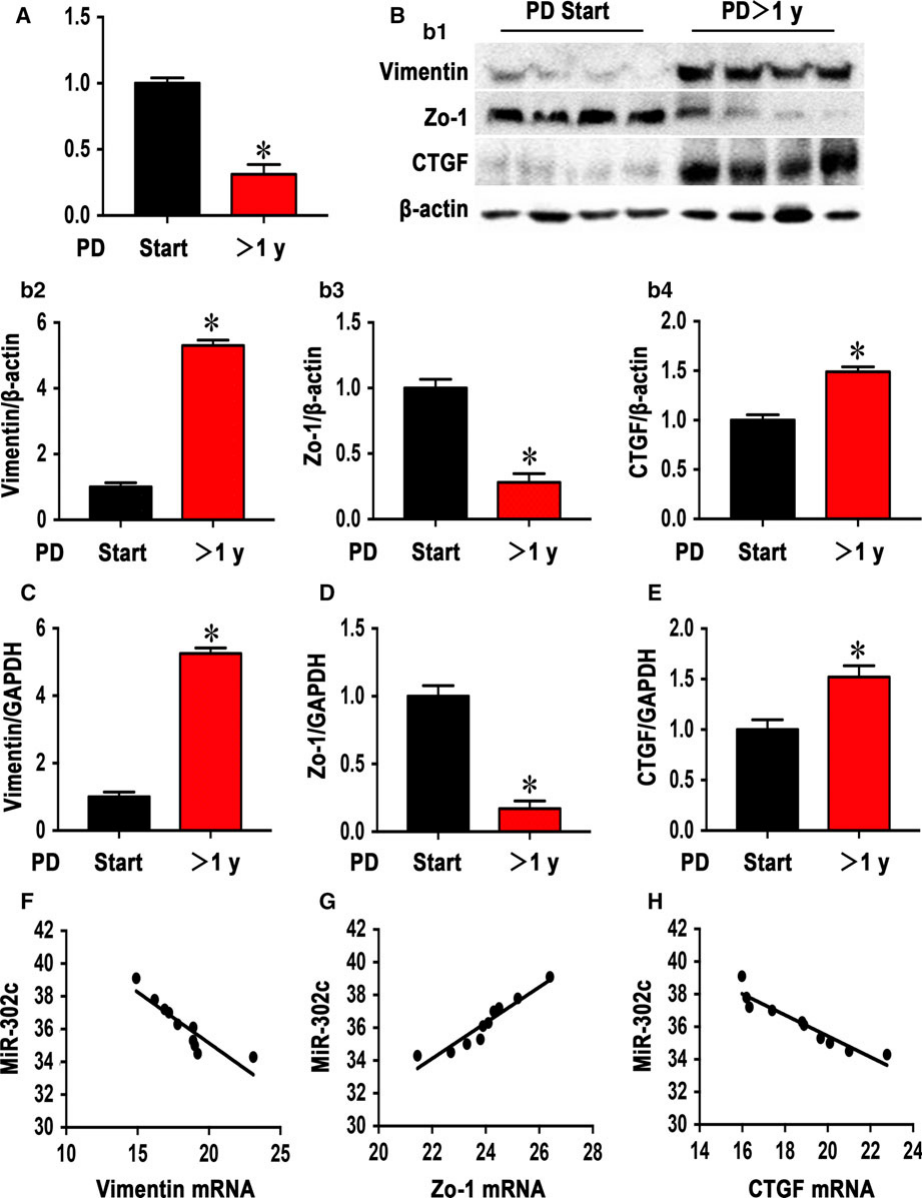
MiR‐302c, MMT‐related factors (vimentin, Zo‐1) and CTGF expression in mesothelial cells isolated from the effluents of patients with PD. (A) Real‐time PCR showed that miR‐302c was significantly down‐regulated in the HPMCs after PD for more than 1 year. Western blot analysis of HPMCs from the effluents of PD patients (B1); the bar graphs (B2‐B4) represent the vimentin, Zo‐1 and CTGF protein band densities relative to β‐actin. The values are the means ± SE (n = 10). Similar results were shown by real‐time PCR (C‐E), vimentin, Zo‐1 and CTGF levels were altered in the PMCs from the effluents of PD patients in the PD start and PD >1 year groups. (F‐H): Pearson correlation analyses of miR‐302c expression with vimentin, Zo‐1 and CTGF mRNA expression. *P < 0.05 vs PD Start

To confirm our previous findings from a miRNA array analysis, we measured miR‐302c and MMT‐related factors in the peritoneal mesothelial cells from the effluents of PD patients. The average duration of dialysis in the PD >1 year group (23.80 ± 13.26 months) was significantly longer than that in the PD start group (1.70 ± 0.82 months). Other clinical characteristics, including sex, age, BMI and D/PCr (4 hours) in the peritoneal equilibrium test, were not significantly different (Table 1). We found that there was a marked reduction in miR‐302c levels with prolonged dialysis (Figure 1A), as well as increased vimentin levels (Figure 1B2 and C) and reduced Zo‐1 levels (Figure 1B3 and D), CTGF expression was also up‐regulated with time (Figure 1b4 and E). Pearson's correlation analyses (Table 2) showed that miR‐302c level was negatively correlated with the mRNA expression of vimentin (*r* = −0.887, Figure 1F) and CTGF (*r* = −0.840, Figure 1H), and positively correlated with the mRNA expression of Zo‐1 (*r* = 0.873, Figure 1G).

**Table 1:** Clinical characteristics of patients in each group

	PD start group	PD > 1 year group	*P* value
Total number of patients (n)	10	10	
Males (n)	5	5	
Females (n)	5	5	>0.05
Age (years)	44.0 ± 13.70	43.20 ± 11.20	>0.05
BMI (kg/m^2^)	21.93 ± 4.37	21.15 ± 3.62	>0.05
Dialysis duration (months)	23.80 ± 13.26	1.70 ± 0.82	<0.01
Peritoneal equilibrium test			
D/PCr (4 h)	0.56 ± 0.08	0.56 ± 0.10	>0.05
High (n)	0	0	
High average (n)	2	2	
Low average (n)	5	6	
Low (n)	3	2	

**Table 2:** Pearson correlation analysis between miR‐302c and MMT‐related indicators in the PD group

Indicators	miR‐302c	
	*r*	*P*
Vimentin	−0.887	0.001
Zo‐1	0.873	0.001
CTGF	−0.840	0.002

### 3.2 MiR‐302c was down‐regulated during peritoneal fibrosis in a mouse model of PD

**Figure 2:**
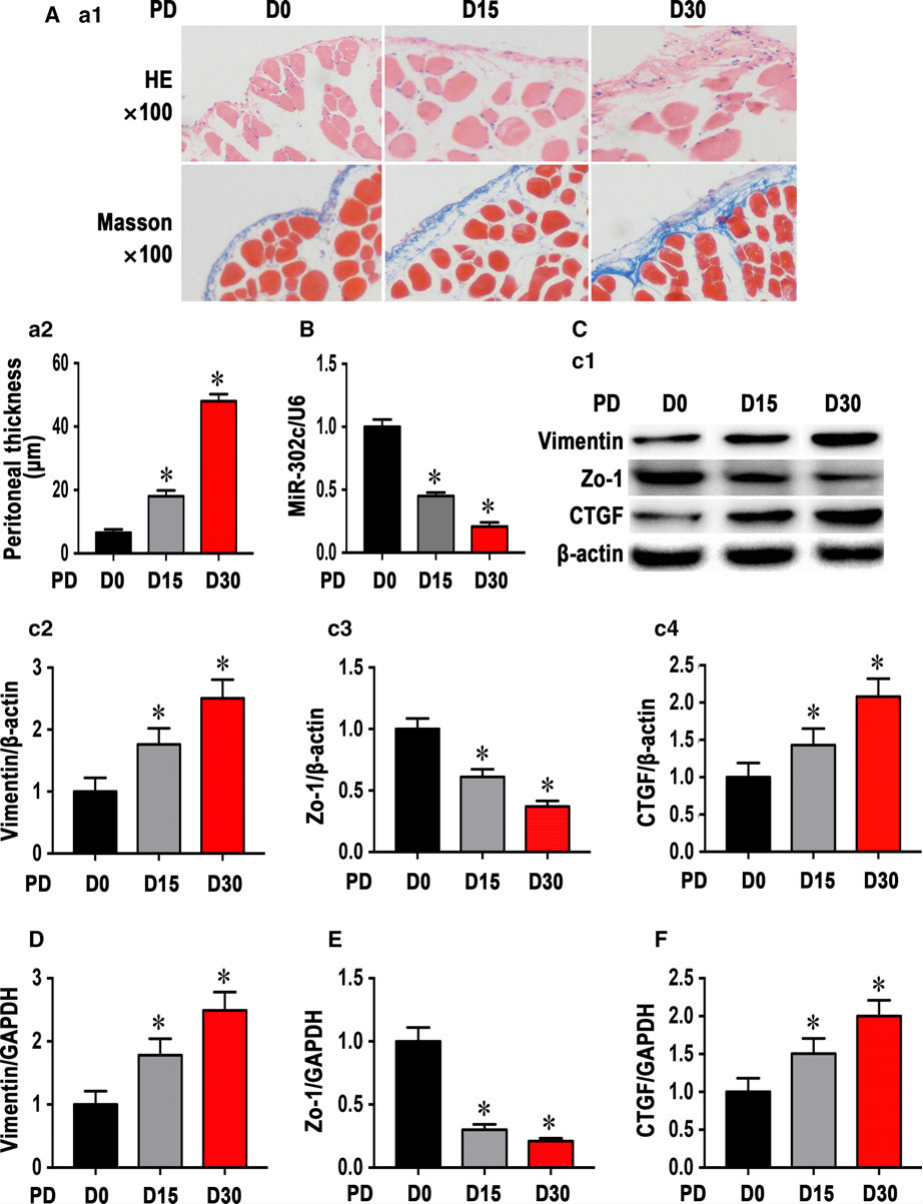
MiR‐302c was down‐regulated in the peritoneum of PD mouse with EMT and fibrosis development over time. (A1) HE and Masson's trichrome staining showed that the submesothelial compact zone thickened with a longer PD time (100× magnification). (A2) The graph indicates the quantification of the peritoneal thickness of the three groups. (B) Real‐time PCR shows that miR‐302c levels in the peritoneum were significantly lower in the PD mice in the D30 and D15 group than in those in the D0 group. Western blot analysis of peritoneum from PD mice (C1); the bar graphs (C2‐C4) represent the vimentin, Zo‐1 and CTGF protein band densities relative to β‐actin. The values are the means ± SE (n = 6). Similar results were shown by real‐time PCR (D‐F). *P < 0.05 vs D0 group

We set up a PD mouse model according to our previous research and found that the submesothelial compact zone of the peritoneum was thickened with an extended time of PD (Figure 2A), and real‐time PCR showed that miR‐302c was down‐regulated in the peritoneum of mice from the D30 group compared with the D0 group (Figure 2B). Western blot analyses showed that MMT was present with increased vimentin (Figure 2C2) and decreased Zo‐1 levels (Figure 2C3), and the CTGF expression was up‐regulated with time (Figure 2C4). Similar results were also seen in their mRNA expression levels (Figure 2D‐F). These results suggested a correlation between miR‐302c and MMT.

### 3.3 MiR‐302c overexpression protected the peritoneum from PD‐induced peritoneal fibrosis in vivo

**Figure 3:**
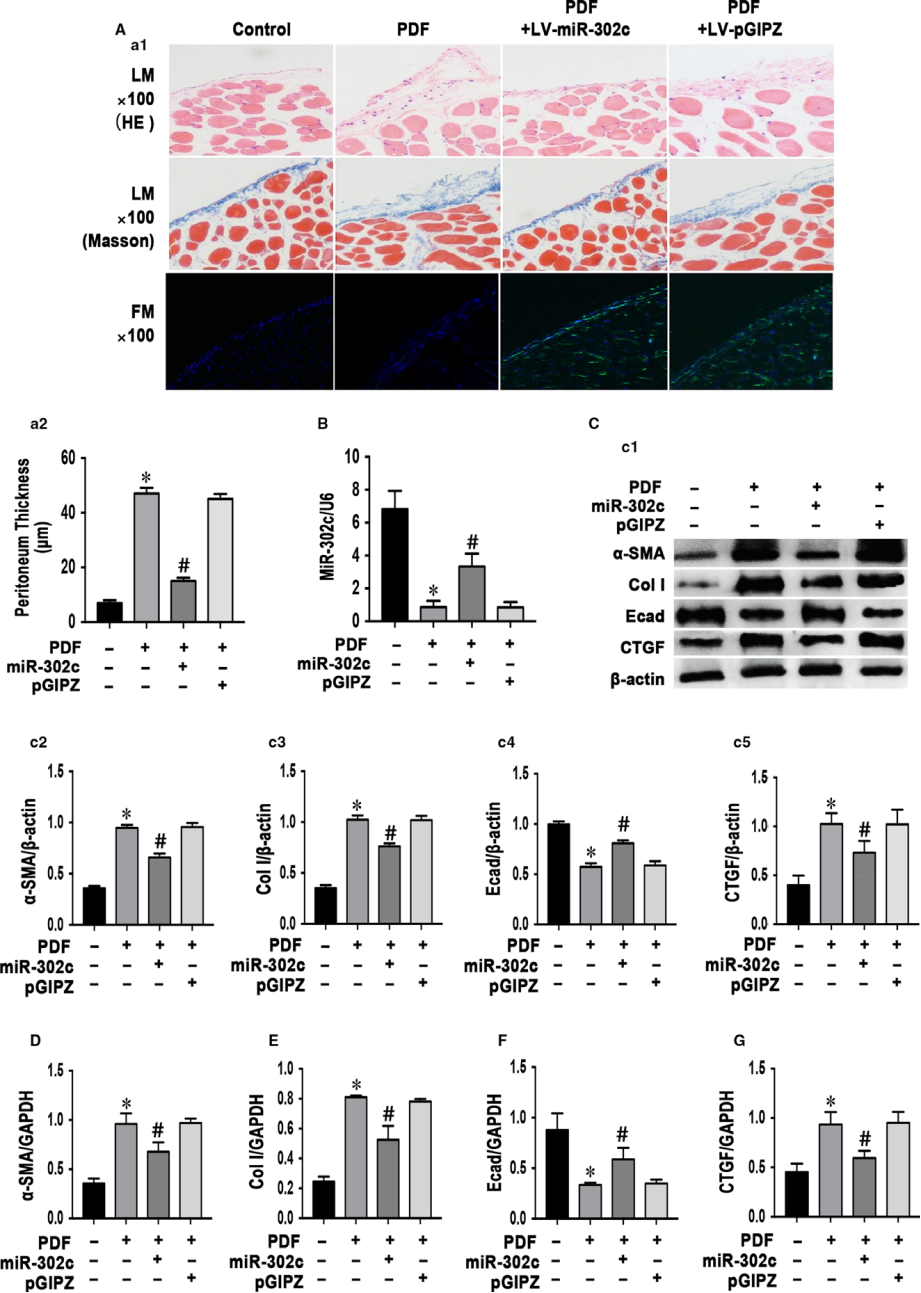
MiR‐302c transfection protects the peritoneal membrane from PD‐induced fibrosis. (A1) Under a light microscope, HE and Masson's trichrome staining showed that the overexpression of miR‐302c alleviates PD‐induced peritoneal fibrosis (100× magnification). Under a fluorescence microscope, it was found that obvious green fluorescence in the mesothelial cells and submesothelial compact zone of the peritoneum with the transfection of LV‐mmu‐miR‐302c or LV‐pGIPZ. (A2) The graph indicates the quantification of the peritoneal thickness in the four groups. (B) Real‐time PCR shows that the peritoneum transfected with LV‐mmu‐miR‐302c overexpressed miR‐302c compared to other groups. Western blot analysis (C1) of the overexpression of miR‐302c inhibiting the PD‐induced up‐regulation of α‐smooth muscle actin (α‐SMA) and collagen I and the down‐regulation of E‐cadherin in the peritoneal membrane at the 30th day. In addition, the up‐regulation of CTGF during PD was reversed by the overexpression of miR‐302c, and the bar graphs (C2‐C5) represent the α‐SMA, collagen I, E‐cadherin and CTGF protein band densities relative to β‐actin. The values are the mean ± SE (n = 5). Similar results were shown by real‐time PCR (D‐G), *P < 0.05 vs Control group. ^#^ *P* < 0.05 vs PDF group. Col I: collagen I; E‐cad: E‐cadherin

To determine the functional role of miR‐302c in a mouse PD model of peritoneal fibrosis, we used LV‐mmu‐miR‐302c to transfect mouse peritoneum and LV‐pGIPZ as a control. It was found that obvious green fluorescence in the mesothelial cells and submesothelial compact zone of the peritoneum with the transfection of LV‐mmu‐miR‐302c or LV‐pGIPZ (Figure 1A1). Meanwhile, miR‐302c was obviously up‐regulated in PD mice transfected with LV‐mmu‐miR‐302c compared with those that received only PD fluid (Figure 3B). This up‐regulation resulted in a significant attenuation of peritoneal fibrosis, as demonstrated by HE and Masson's trichrome staining (Figure 3A1). In the control group, the peritoneal tissues were almost normal and had no thickening of the submesothelial compact zone. Compared with that in the control group, the submesothelial compact zone in the peritoneum of the PDF group was significantly thicker and enriched with numerous cells. However, the intraperitoneal injection of LV‐mmu‐miR‐302c suppressed the thickening of the submesothelial compact zone (Figure 3A2). Moreover, the down‐regulation of E‐cadherin and the up‐regulation of α‐SMA and collagen I during PD were alleviated (Figure 3C2‐C4 and D‐F). The up‐regulation of CTGF induced by PDF was also alleviated by miR‐302c overexpression (Figure 3C5 and G).

### 3.4 MiR‐302c is down‐regulated by TGF‐β1 during MMT in vitro

**Figure 4:**
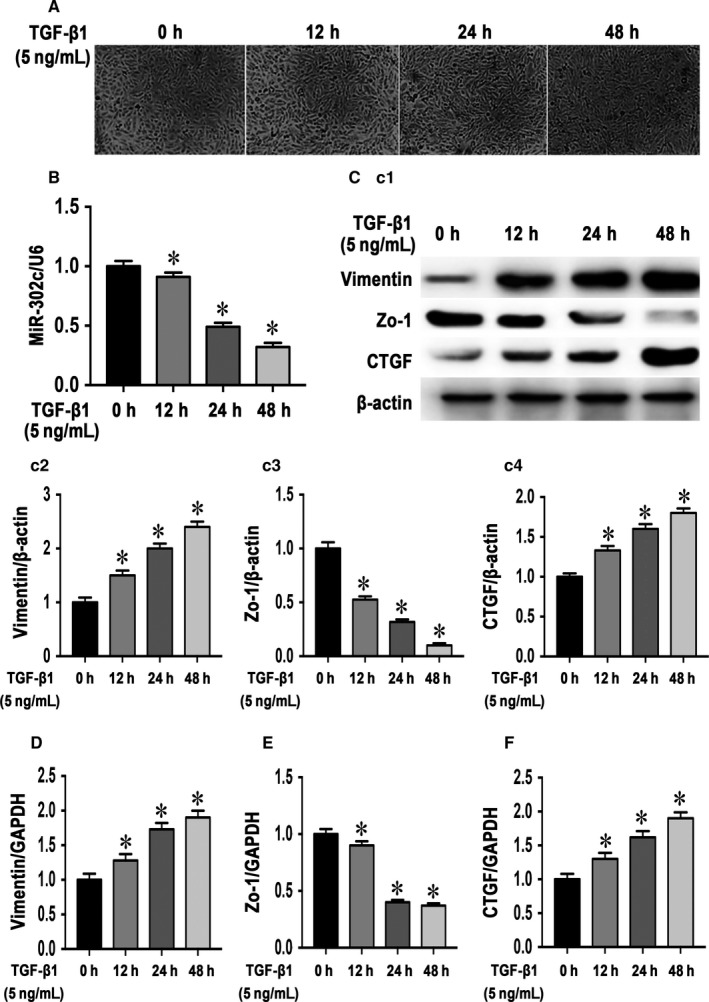
MiR‐302c was down‐regulated during TGF‐β1‐induced MMT in HMrSV5 cells. (A) Morphological changes in HMrSV5 cells after 5 ng/mL TGF‐β1 treatment for 0, 12, 24 and 48 h. (B) MiR‐302c down‐regulation in HMrSV5 cells in a time‐dependent manner. Western blot analysis (C1) of HMrSV5 cells; the bar graphs (C2‐C4) represent vimentin, Zo‐1 and CTGF protein band densities relative to β‐actin. The values are the mean ± SE (n = 3). Similar results were shown by real‐time PCR (D‐F). *P < 0.05 vs 0 h

MMT has been proven to be an important mechanism in the pathogenesis of peritoneal fibrosis during PD. We next examined the expression of miR‐302c in TGF‐β1‐induced MMT in HMrSV5 cells. The incubation of HMrSV5 cells with 5 ng/mL TGF‐β1 for 12, 24 and 48 hours resulted in a remarkable time‐dependent fibroblast‐like morphological changes in HMrSV5 cells (Figure 4A). The expression of miR‐302c was also down‐regulated in a time‐dependent manner (Figure 4B). These morphological changes in HMrSV5 cells were associated with the up‐regulation of vimentin (Figure 4C2 and D) and the down‐regulation of Zo‐1 (Figure 4C3 and E). In addition, the expression of CTGF was also up‐regulated with time as in the in vivo experiment above (Figure 4C4 and F).

### 3.5 MiR‐302c overexpression attenuates TGF‐β1‐induced MMT and fibrogenesis in HPMCs through down‐regulation of CTGF

**Figure 5:**
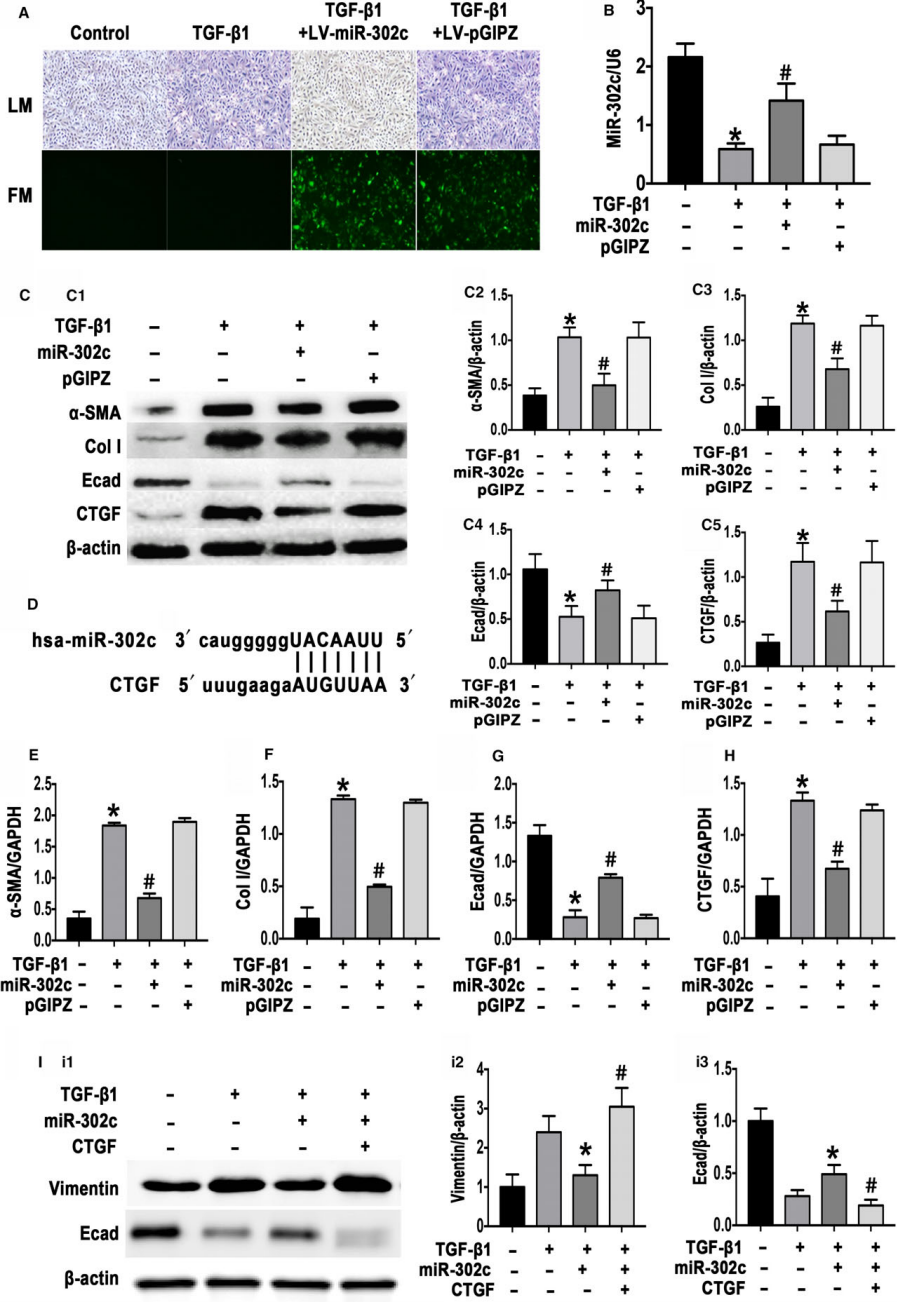
Overexpression of miR‐302c by lentivirus transfection inhibited TGF‐β1‐induced MMT and peritoneal fibrosis in HMrSV5 cells through down‐regulation of CTGF. (A) LV‐hsa‐miR‐302c was used to stably transfect HMrSV5 cells, and GFP was examined under a fluorescence microscope; the overexpression of miR‐302c alleviated the fibroblast‐like morphological changes in HPMCs. (B) Real‐time PCR indicated that LV‐hsa‐miR‐302c‐transfected HMrSV5 cells overexpressed miR‐302c compared to the other groups. Western blot analysis (C1) showed that the overexpression of miR‐302c alleviated TGF‐β1‐induced MMT and fibrosis and prevented the down‐regulation of E‐cadherin and the up‐regulation of α‐smooth muscle actin (α‐SMA) and collagen I in HMrSV5 cells; moreover, the up‐regulation of CTGF induced by TGF‐β1 was reversed by the overexpression of miR‐302c, the bar graphs (C2‐C5) represent the α‐SMA, collagen I, E‐cadherin and CTGF protein band densities relative to β‐actin. The values are the means ± SE (n = 3). Similar results were shown by real‐time PCR (E‐H); (D) Location of the predicted miR‐302c target site in the CTGF 3′UTR, as assessed by biological analysis (www. microrna.org). (I) Vimentin and E‐cadherin protein expression in HPMCs treated with TGF‐β1, LV‐miR‐302c and recombinant CTGF by western blot analysis. *P < 0.05 vs Control group. ^#^ *P* < 0.05 vs TGF‐β1 group. Col I: collagen I; Ecad: E‐cadherin

To determine the functional role of miR‐302c in the process of MMT and peritoneal fibrosis, we transfected HMrSV5 cells with LV‐hsa‐miR‐302c and then incubated them with 5 ng/mL TGF‐β1 for 48 hours. We analysed miR‐302c gene expression by detecting the green fluorescent protein (GFP) under a fluorescence microscope; we found that miR‐302c was stably transfected in HMrSV5 cells (Figure 5A), and this result was further confirmed by the real‐time PCR analysis of miR‐302c expression (Figure 5B). The overexpression of miR‐302c alleviated the fibroblast‐like morphological changes in HPMCs (Figure 5A). Both western blot and real‐time PCR analyses revealed that the protective effect of miR‐302c on peritoneal fibrosis was associated with the blockade of MMT and fibrosis, which was manifested by preventing the down‐regulation of the epithelial marker E‐cadherin (Figure 5C4 and G) and the up‐regulation of the mesenchymal markers α‐SMA (Figure 5C2 and E) and collagen I (Figure 5C3 and F). Moreover, the up‐regulation of CTGF was also reversed by the overexpression of miR‐302c (Figure 5, C5 and H), suggesting that the protective effects of miR‐302c against MMT and peritoneal fibrosis may be associated with the inhibition of CTGF. We searched biological analysis software (www.microrna.org) and found that miR‐302c has a biding site in the 3′UTR of CTGF genes (Figure 5D), suggesting that miR‐302c could regulate the expression of CTGF.

To clarify whether miR‐302c modulates TGF‐β1‐induced MMT through down‐regulation of CTGF, recombinant CTGF protein was used to overexpression of CTGF in HPMCs in vitro. Western blot assay shown that LV‐hsa‐miR‐302c transfection significantly inhibited the up‐regulation of vimentin protein and down‐regulation of E‐cadherin induced by TGF‐β1 in HPMCs. However, this effect was partially diminished by co‐treatment with recombinant CTGF protein (Figure 5I), suggesting that miR‐302c may play a protective role in MMT of HPMCs and peritoneal fibrosis through CTGF.

## 4 DISCUSSION

Recently, signalling pathways involved in the pathogenesis of peritoneal fibrosis in PD have been studied, these include TGF‐β1‐induced signalling pathways and Toll‐like receptor ligands‐induced signalling pathways.^24^, ^25^, ^26^, ^27^ As the main factor controlling fibrosis in all organs, TGF‐β1 has been demonstrated to play a central role in MMT. However, the precise mechanism by which TGF‐β1 induces MMT in PD remains unclear. In this study, we demonstrated that the expression of miR‐302c was decreased in both PD patients and a human peritoneal cell line treated with TGF‐β1; additionally, miR‐302c was negatively correlated with MMT and peritoneal fibrosis. The overexpression of miR‐302c ameliorated both the peritoneal fibrosis in the PD mouse model and TGF‐β1‐induced MMT in the human peritoneal cell line, as well as inhibited the increase in CTGF levels. These data suggest that there is a new TGF‐β1/miR‐302c/CTGF pathway that may play a significant role in the process of MMT and fibrosis in PD.

An emerging body of evidence suggests that miRNAs play an important role in the growth and metastasis of tumour cells via regulating the TGF‐β‐induced MMT process.^28^, ^29^, ^30^, ^31^ A previous study has demonstrated that TGF‐β can increase Col1a2 mRNA levels through up‐regulating miR‐192 in the mesangial cells of diabetic mice.^32^ Moreover, a study of the MMT process in tubular epithelial cells showed that the level of let‐7d miRNA was significantly reduced by TGF‐β stimulation, and the overexpression of let‐7d could suppress TGF‐β‐induced MMT and renal fibrogenesis.^33^ With respect to peritoneal fibrosis, our previous studies have demonstrated that miRNA589 is decreased and negatively regulates MMT in cultured HPMCs treated with TGF‐β1,^34^ and miR‐129‐5p modulates MMT by targeting SIP1 and SOX4 during PD^8^; moreover, other miRNAs, such as miR‐30a, miR‐30b and miR‐29b, have been reported to regulate TGF‐β1‐induced MMT and peritoneal fibrosis.^6^, ^25^, ^35^ All these studies suggest that miRNAs may play a key role in peritoneal fibrosis during PD.

In clinical settings, PD effluent markers were used to assess peritoneal functionality and morphology. Established effluent biomarkers include cancer antigen 125 (CA125), VEGF, hyaluronan (HA), interleukin‐6 (IL‐6) and tumour necrosis factor (TNF‐α).^36^, ^37^, ^38^ In various studies, PD effluent was also used as a source of biomarkers for the early detection of long‐term alterations in the peritoneum and peritoneal fibrosis. In our previous study, aberrant miRNA levels associated with PD were observed using a miRNA array analysis of cultured PMCs from the effluent of PD patients. MiR‐302c was found to be down‐regulated nearly threefold in patients undergoing PD for more than 1 year when compared with patients who had recently started PD. We further demonstrate that the expression levels of miR‐302c are decreased in both PD patients and a cultured human peritoneal mesothelial cell line treated with TGF‐β1; however, miR‐302c expression is negatively correlated with the development of MMT and fibrosis during PD (Figures 1 and 2).

Lentivirus is a subtype of retrovirus and is originally developed from human immunodeficiency virus (HIV). Lentiviral vectors share the features of other retroviral vectors as a highly effective gene delivery system, which could mediate stable gene transfer in non‐dividing cells and would not trigger a potent immunogenic or inflammatory response. It was delineated that E‐cadherin expression obviously down‐regulated and cell junction disappeared with infection of p38‐shRNA mediated by lentiviral vectors to mesothelial cells.^39^ In our study, lentivirus vectors were employed and successfully overexpressed miR‐302c in PD mice peritoneum with the observation of green fluorescence. Encouragingly, with the increased level of miR‐302c, MMT‐related genes and proteins (E‐cadherin and α‐SMA) expression dramatically ameliorated in PD mice. Moreover, the expression of CTGF and collagen I expression obviously up‐regulated, suggesting overexpression of miR302c significantly alleviated PD‐related peritoneal fibrosis (Figure 3). The 3′UTR of the CTGF gene included a predicted binding site for miR‐302c (Figure 5) according to biological analysis software (www.microrna.org); this finding suggests that CTGF may be a target gene of miR‐302c. We also observed that the overexpression of miR‐302c in HPMCs reversed the TGF‐β1‐induced MMT phenotypic transformation and the increase in CTGF levels. These data suggest that miR‐302c plays a significant role in regulating the TGF‐β1/CTGF pathway in the process of MMT and fibrosis during PD.

In our study, we observed that the expression of miR‐302c is significantly down‐regulated in both a cultured human peritoneal mesothelial cell line treated with TGF‐β1 and peritoneal tissue of a PD mouse model. These changes were associated with decreased expression levels of E‐cadherin and increased expression levels of α‐SMA and collagen I. On the other hand, the overexpression of miR‐302c notably reversed the effects of TGF‐β1‐induced MMT, thus regulating the expression of MMT‐related genes and proteins and ameliorating the phenotype changes in HPMCs and the fibrosis extent in the peritoneal tissues of PD mice. These data suggest that miR‐302c acts as a negative modulator of the MMT and fibrosis that are induced by TGF‐β1 during PD.

There are many transcription factors and pathways involved in the TGF‐β1‐induced MMT process; these include ZEB1, SIP1,^40^ snail, slug,^41^, ^42^ SOX4,^43^ Jagged/Notch^44^ and the Wnt/β‐catenin^45^ and TGF‐β/smad pathways.^35^ To clarify the molecular mechanism by which miR‐302c modulates TGF‐β1‐induced MMT in PD, we investigated CTGF, which is a downstream mediator of TGF‐β1. Many studies have demonstrated the pro‐sclerotic role of CTGF in peritoneal fibrosis during PD because the dialysate CTGF concentration could be a biomarker for peritoneal fibrosis. Studies have also reported that using tamoxifen and tanshinone IIA to reduce CTGF expression can ameliorate peritoneal fibrosis.^46^, ^47^

Our previous study demonstrated the positive correlations between the levels of CTGF, TGF‐β1 and FN proteins and the collagen thickness in PD rats. These studies indicated that CTGF is an important mediator implicated in the pathogenesis of peritoneal fibrosis.

In contrast to the diverse effects of TGF‐β1, CTGF specifically targets the fibrosis pathway and is thus an attractive candidate for inhibiting the damage to the membrane.^48^ Furthermore, increasing the expression levels of CTGF can induce partial MMT in renal tubular cells. This process cannot be blocked by neutralizing anti‐TGF‐β1 antibodies, suggesting that this action is TGF‐β1 independent. From these studies we can infer that CTGF may be a better therapeutic target for peritoneal fibrosis than TGF‐β1.

Our study indicated that the levels of CTGF were significantly increased in both a cultured human peritoneal mesothelial cell line treated with TGF‐β1 and the peritoneal tissue of a PD mouse model. The up‐regulation of CTGF is tightly correlated with the expression of MMT‐related genes and proteins and is negatively correlated with the expression of miR‐302c. In addition, we observed that the overexpression of miR‐302c decreased the CTGF mRNA and protein levels. These data indicate that miR‐302c may exert a protective role in the MMT during PD by regulating the expression of CTGF, and the TGF‐β1/miR‐302c/CTGF pathway may take part in modulating the process of MMT during PD.

However, one study has demonstrated that TGF‐β regulates CTGF through increasing the expression of the miR‐302 cluster.^49^ MiR‐302c decreased the TGF‐β‐induced MMT in renal epithelial cells and the TGF‐β‐induced mesangial production of fibronectin and thrombospondin. These results indicate a feedback loop between TGF‐β and miR‐302c that needs further study in the process of MMT and fibrosis in PD.

In summary, our study suggests that miR‐302c is a vital factor protecting MCs from undergoing TGF‐β1‐induced MMT during PD. We also demonstrated that miR‐302c may inhibit TGF‐β1‐induced MMT by suppressing the expression of CTGF. Therefore, our data provide new information regarding the mechanism of peritoneal fibrosis during PD and may offer a new therapeutic target.

## CONFLICT OF INTEREST

The authors confirm that there are no conflicts of interest.
