# Supplementary material for: MicroRNA‐302c modulates peritoneal dialysis‐associated fibrosis by targeting connective tissue growth factor
Source: J Cell Mol Med. 2019 Jan 28;23(4):2372–83. doi: 10.1111/jcmm.14029 (PMC6433681; doi:10.1111/jcmm.14029)
Supplement: Supplementary file 1 [file JCMM-23-2372-s001.docx]

| Gene name | Primer sequence (5’-3’) |
| --- | --- |
| GAPDH | F: CGACCACTTTGTCAAGCTCA  R: AAAGCTGGGTCCAACTCCTT |
| hsa-VIM | F: TTGAACGCAAAGTGGAATC  R: AGGTCAGGCTTGGAAACA |
| hsa-TJP1 | F: TGGTGTCCTACCTAATTCAACTCA  R: CGCCAGCTACAAATATTCCAACA |
| hsa-COL1A1 | F: CCTGGATGCCATCAAAGTCT  R: TCTTGTCCTTGGGGTTCTTG |
| hsa-ACTA2 | F: CTGAGCGTGGCTACTCCTTC  R: GCCATCTCGTTCTCGAAGTC |
| hsa-CDH1 | F: GAACGCATTGCCACATACAC  R: ATTCGGGCTTGTTGTCATTC |
| hsa-CTGF | F: TTGCGAAGCTGACCTGGAAGAGAA  R: AGCTCGGTATGTCTTCATGCTGGT |
| mmu-TJP1 | F: CGAGGCATCATCCCAAATAAGAAC  R: TCCAGAAGTCTGCCCGATCAC |
| mmu-VIM | F: ACCGCTTTGCCAACTACAT  R: TTGTCCCGCTCCACCTA |
| mmu-COL1A1 | F: CACCCTCAAGAGCCTGAGTC  R: TCCGCTCTTCCAGTCAGAGT |
| mmu-ACTA2 | F: AGACAGCTATGTGGGGGATG  R: CTTTTCCATGTCGTCCCAGT |
| mmu-CDH1 | F: GCACTCTTCTCCTGGTCCTG  R: TATGAGGCTGTGGGTTCCTC |
| mmu-CTGF | F: GGGGAGGGCAATTATAGCAT  R: AAAGCTGGGTCCAACTCCTT |

**Supplementary Table 1. The primer sequences for RT-PCR analysis.**
